# Network Properties Revealed during Multi-Scale Calcium Imaging of Seizure Activity in Zebrafish

**DOI:** 10.1523/ENEURO.0041-19.2019

**Published:** 2019-03-11

**Authors:** Jing Liu, Scott C. Baraban

**Affiliations:** Department of Neurological Surgery and Weill Institute for Neuroscience, University of California, San Francisco, CA 94143

**Keywords:** epilepsy, fast confocal, neuronal networks, synchronization, whole-brain imaging, zebrafish

## Abstract

Seizures are characterized by hypersynchronization of neuronal networks. Understanding these networks could provide a critical window for therapeutic control of recurrent seizure activity, i.e., epilepsy. However, imaging seizure networks has largely been limited to microcircuits *in vitro* or small “windows” *in vivo*. Here, we combine fast confocal imaging of genetically encoded calcium indicator (GCaMP)-expressing larval zebrafish with local field potential (LFP) recordings to study epileptiform events at whole-brain and single-neuron levels *in vivo*. Using an acute seizure model (pentylenetetrazole, PTZ), we reliably observed recurrent electrographic ictal-like events associated with generalized activation of all major brain regions and uncovered a well-preserved anterior-to-posterior seizure propagation pattern. We also examined brain-wide network synchronization and spatiotemporal patterns of neuronal activity in the optic tectum microcircuit. Brain-wide and single-neuronal level analysis of PTZ-exposed and 4-aminopyridine (4-AP)-exposed zebrafish revealed distinct network dynamics associated with seizure and non-seizure hyperexcitable states, respectively. Neuronal ensembles, comprised of coactive neurons, were also uncovered during interictal-like periods. Taken together, these results demonstrate that macro- and micro-network calcium motifs in zebrafish may provide a greater understanding of epilepsy.

## Significance Statement

Monitoring the dynamic activities in large-scale neuronal networks is critical to understanding seizure initiation and propagation. Here, we used well-established larval zebrafish seizure protocols and fast confocal imaging of genetically encoded calcium indicator (GCaMP)-expressing fish to investigate the epileptic network properties at brain-wide and single-cell levels. We revealed the rapid propagation of seizure activity from anterior-to-posterior brain regions in zebrafish CNS. We also showed that micro-ensembles of neuronal subpopulations are active during interictal-like periods in a manner similar to that seen in human electrophysiology data sets. Our findings demonstrate that these non-invasive optical imaging approaches will advance our understanding of the network basis underlying seizures and facilitate the development of methods to suppress these events.

## Introduction

Synchronization of neuronal networks is a commonly observed event in the brain and underlies pathologic generation and propagation of epileptic seizures. Although electrophysiological features of an individual seizure event are well characterized using local field potential (LFP) and/or single-unit electrophysiological recordings (Grasse et al., 2013; Park et al., 2013; Schmidt et al., 2014; Truccolo et al., 2014; Weiss et al., 2016), these approaches do not capture brain-wide spatiotemporal network dynamics underlying these events. Efforts to image these networks have primarily been limited to small microcircuits contained within brain slice preparations or imaging “windows” in head-restrained mice (Trevelyan et al., 2006; Muldoon et al., 2013, 2015; Grosser et al., 2014; Kuzum et al., 2014; Hongo et al., 2015; Lillis et al., 2015; Rossi et al., 2017; Wenzel et al., 2017; Liou et al., 2018). Recent advances in imaging technology combined with stable expression of genetically encoded calcium indicators (GCaMPs; Tian et al., 2009; Akerboom et al., 2012; Chen et al., 2013) have led to a revolution in brain-wide activity mapping. GCaMPs are employed in a range of model organisms; among these, larval zebrafish (*Danio rerio*) offer an unparalleled combination of higher vertebrate relevance (including human), optical transparency, and a small brain size (comprising ∼100,000 neurons) allowing spatial coverage of multiple brain regions. Moreover, the availability of transgenic zebrafish, in which GCaMPs are under the control of neuronal *elavl3* or *NeuroD* promoters has greatly facilitated the potential for assessing neural networks. Indeed, brain-wide imaging in GCaMP-expressing zebrafish has already been used to monitor spatiotemporal patterns of neural activity during fictive swimming (Ahrens et al., 2013a; Thiele et al., 2014), motor adaptation (Ahrens et al., 2012), visual processing (Akerboom et al., 2012; Muto et al., 2013), and prey capture (Muto and Kawakami, 2013; Semmelhack et al., 2014). During this time, zebrafish have also emerged as a valuable vertebrate model for neurologic disease, particularly for epilepsy (Hortopan et al., 2010; Ramirez et al., 2012; Stewart et al., 2014; Grone and Baraban, 2015; Martín-Jiménez et al., 2015; Griffin et al., 2017). Combining these advances, it is now possible to non-invasively monitor activity throughout the zebrafish nervous system during an epileptic seizure event.

Brain-wide imaging during seizures, in contrast to invasive electrophysiological recordings, increases the likelihood that network patterns will be discovered. To this end, we combined well-established acute seizure (pentylenetetrazole; PTZ; Baraban et al., 2005; Baxendale et al., 2012; Afrikanova et al., 2013; Buenafe et al., 2013; Orellana-Paucar et al., 2013; Rahn et al., 2014; Siebel et al., 2015; Barbalho et al., 2016; Copmans et al., 2018) and non-seizure hyperexcitability (4-aminopyridine; 4-AP) models (Ellis et al., 2012; Kumar et al., 2016; Cassar et al., 2017; Winter et al., 2017) with spinning disk confocal microscopy, enabling fast (20–30 fps) *in vivo* imaging of network events. Imaging studies were performed at brain-wide and single-cell resolution levels. As anesthetics can modify neuronal activity (Greenberg et al., 2008), experiments were done using “awake” agarose-embedded larval zebrafish paralyzed with pancuronium to reduce movement artifact. Simultaneous measurements of LFPs were used to confirm seizure activity. By analyzing brain-wide changes in calcium dynamics in an acute seizure model, we revealed neuronal populations in telencephalon initiating ictal-like seizure events that rapidly generalized to all CNS regions. The spatiotemporal patterns of calcium signal showed a rapid propagation of seizure activity from anterior-to-posterior brain regions. Subsequent analysis of spontaneous activity patterns established a clear pattern of synchronization during ictal-like seizure events consistent with a long-standing notion that seizures are a manifestation of hypersynchronous neural networks. Automated calcium signal processing and spike detection confirmed this high degree of synchronization at a single-neuron level. Finally, we showed that neuronal ensembles (Tao et al., 2011; Truccolo et al., 2011, 2014; Miller et al., 2014) are active during interictal-like periods. We suggest that these non-invasive optical imaging approaches could provide a more comprehensive understanding of complex neuronal networks involved in the generation and propagation of seizures.

## Materials and Methods

### Zebrafish lines

All procedures followed National Institute of Health and the University of California, San Francisco guidelines and were approved by the Institutional Animal Care and Use Committee. Calcium imaging experiments were performed on the *Tg(neurod1:GCaMP6f)* line (Rupprecht et al., 2016) in the nacre (*mitfa^-/-^*) background (kindly provided by Dr. C. Wyart from Institut du Cerveau et de la Moelle Épinière, Paris) on 5–6 d postfertilization (dpf). Adult zebrafish were maintained at 28°C on a 14/10 h light/dark cycle following standard methods. Larvae were raised in embryo media consisting of 0.03% Instant Ocean (Aquarium Systems, Inc.) and 0.0002% methylene blue in reverse osmosis-distilled water. At 5–6 dpf, zebrafish have not yet experienced sexual differentiation (Liew and Orbán, 2014).

### Calcium imaging and electrophysiology

**Figure 1.**
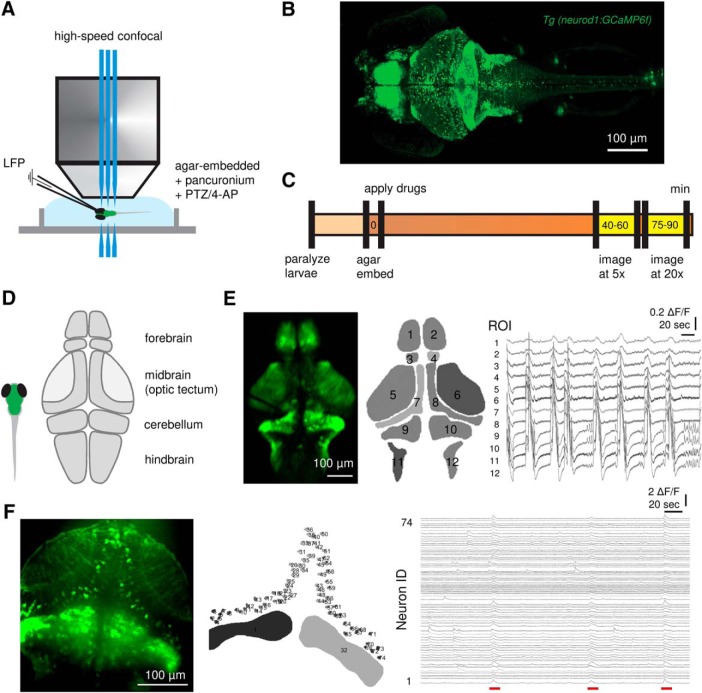
High-speed confocal calcium imaging at different scales. ***A***, Experimental setup. Simultaneous LFP recording and fast confocal imaging (20–30 fps) in agar-embedded larval zebrafish exposed to pancuronium (300 µM) with PTZ (10 mM) or 4-AP (4 mM). ***B***, Representative high-resolution imaging of neurod1:GCaMP6f expressed larval zebrafish on 5–6 dpf. ***C***, Experimental workflow. Recordings were obtained ∼40 min after drug application, and 5× and 20× objectives were used for whole-brain and neuron-level imaging, respectively. ***D***, Schematic illustration depicting sub-regions of the larval zebrafish brain. **E,** ROIs and representative calcium traces (ΔF/F) of PTZ-induced ictal-like events; 1, 2: pallium; 3, 4: habenula; 5, 6: neuropil; 7, 8: SPV; 9, 10: cerebellum; 11, 12: hindbrain. ***F***, Neuronal microcircuits within optic tectum and representative calcium traces of individual SPV neurons with PTZ-induced ictal-like event break-ins (underlined in red). Cerebellum was included as an indicator of ictal-like events that involve all brain regions. Scale bars as indicated in figure.

Zebrafish larvae were paralyzed in 300 µM pancuronium (Abcam), and then immobilized in a drop of 2% low-melting point agarose in a recording chamber (Fig. 1*A*). The recording chamber was placed on the stage of a Zeiss Axiocam upright microscope using a 5× or 20× objective (for whole-brain and neuron-level imaging, respectively), Yokogawa CSU-X1 Spinning Disk Confocal and a 470-nm laser light source (3i LaserStack). The recording chamber was filled with embryo media containing pancuronium (300 µM). Epileptiform activities were induced by bath application of PTZ (10 mM; Sigma-Aldrich) or 4-AP (4 mM; Sigma-Aldrich), and the fish were allowed to establish consistent epileptiform activities for at least 40 min before imaging studies were initiated (Fig. 1*C*). Images of 512 × 512 pixels were acquired at 20–30 Hz with an EMCCD camera (Photometrics Evolve). Multiple 5-min recordings (6000–9000 frames) were acquired for each experiment using SlideBook software (3i Intelligent Imaging Innovations). To record LFPs, a glass microelectrode was placed under visual guidance in the optic tectum or cerebellum. Electrodes were filled with 2 M NaCl, and electrical activity was recorded using an Axopatch 1D amplifier (Molecular Devices). Voltage records were low-pass filtered at 1 kHz (–3 dB, 8-pole Bessel), high-pass filtered at 0.1 Hz, digitized at 10 kHz using a Digidata 1520 A/D interface, and stored on a PC computer running Axoclamp software (Molecular Devices).

### Image analysis

The raw images were first processed to correct motion drift using the Template Matching image registration plugin for ImageJ. Regions of interest (ROIs) for brain regions and single neurons were manually segmented with the ROI manager of ImageJ (Fig. 1*E*,*F*). Corrected image stacks and ROI segmentation file were then imported to MATLAB (MathWorks) for calcium fluorescence signal extraction and analysis using the FluroroSNNAP software (Patel et al., 2015). The calcium fluorescence signal for each brain region/neuron was obtained by averaging all pixels within the ROI. The fluorescence changes (ΔF/F) were calculated by subtracting each data point with the mean of the lower 50% of values within previous 10-s sliding window and normalized to the mean of the lower 50% of values within previous 10-s sliding window. Automated event detection was performed with the template-matching algorithm (Schultz et al., 2009) included in the FluroroSNNAP. A time-varying correlation coefficient between fluorescence trace and calcium transient templates (from the event wave form library) was calculated. Fluorescence transients with amplitude ΔF/F > 0.1 and correlation coefficient >0.85 were identified as events. Local maxima of the correlation coefficient trace identify the timestamps of calcium events. Since the algorithm may sometimes give errors, the event train was then manually corrected by deleting falsely detected events and adding events missed by the algorithm.

To determine the correlation between simultaneous calcium activity and LFP, the amplitude and duration of ictal-like seizure events in PTZ-treated zebrafish were evaluated. The amplitude is the absolute value of the maximum event peak, and the duration is the interval between the first and last points of the trace leaving and returning the baseline.

### Network synchronization

Whole-brain network synchronization was evaluated with the network analysis module of FluroroSNNAP.

Based on the instantaneous phase of the fluorescence trace of each brain region, a pair-wise phase synchronization matrix was generated to identify patterns of network activity (Patel et al., 2015). According to the matrix, an eigenvalue-based algorithm was applied to calculate the global synchronization index which range from 0 (non-coordinated activity) to 1 (completely synchronized activity). For mathematical details see (Li et al., 2007). Briefly, the eigenvalues of the synchronization matrix were calculated and then normalized to generate an array of synchronization indices. The largest entry in this array is the global synchronization index. If the time series are fully uncorrelated, all elements in the synchronization matrix would be 0, and the eigenvalue would be 0, resulting a global synchronization index equal to 0. Alternatively, if the network was completely synchronized, all elements in the synchronization matrix would be 1, and the maximal eigenvalue would be equal to the number of time series with other eigenvalues falling to zero, resulting a global synchronization index equal to 1.

### Seizure propagation

To determine the recruitment timing of each brain region to an ictal-like event, we took the time derivative (slope) of the fluorescence trace (smoothed by taking moving average of 50 data points), and used the local maxima (the fastest change in fluorescence) to identify the onset time point (Wenzel et al., 2017). Each brain region has two data points corresponding to the left and right counterparts. We then calculated the relative lag of each brain region with respect to the earliest onset within an ictal-like event and normalized it to the interval between the earliest onset and the latest onset. Normalized relative lags within an ictal-like event were divided into three quantile groups (first third, early; second third, middle; third third, late), and the probability of each brain region falling into each quantile group was calculated.

### Detection of ensemble events

Ensemble events were defined as coactivation of a group of neurons in which a statistically significant number of neurons are active compared with surrogate datasets. We used a sliding window to generate a time series of coactivation of neurons by counting the number of events within a 0.5-s (10 frames) window. The binary event data were shuffled 2000 times within neurons, and the sliding window counting was performed. Frames with an observed number of coactive neurons >99.9% of all surrogate values (*p* < 0.001) were identified as highly active frames with ensemble event. To analyze the spatial extent of neuronal ensembles, the distances between the ensemble centroid and individual neurons within the ensemble were calculated using standard Euclidian distances.

### Experimental design and statistical analysis

We used one-way ANOVA with Holm–Sidak *post hoc* analysis for multiple variable comparison, and χ^2^ test for onset timing distribution analysis, which were performed in Prism (GraphPad Software). Correlation coefficients (*R* ^2^) between calcium and electrical signals were calculated in MATLAB (MathWorks). Detection of neuronal ensembles was performed in MATLAB by comparing with surrogate datasets (see Materials and Methods). Individual analyses are described in Results.

## Results

To study synchronized neural networks, we used two well-characterized pharmacological models employing bath application of PTZ (10 mM) or 4-AP (4 mM). These acute chemoconvulsant models work through different mechanisms: (1) PTZ acts as a GABA_A_ receptor antagonist to disinhibit network activity (Baraban et al., 2005; Baxendale et al., 2012; Afrikanova et al., 2013; Buenafe et al., 2013; Orellana-Paucar et al., 2013; Rahn et al., 2014; Siebel et al., 2015; Barbalho et al., 2016; Copmans et al., 2018) and (2) 4-AP is a potassium channel blocker that enhances neuronal firing activity (Ellis et al., 2012; Kumar et al., 2016; Cassar et al., 2017; Winter et al., 2017). Together, these acute models provide a strategy to study recurrent spontaneous seizure events (PTZ) and non-seizure hyperexcitability (4-AP), respectively.

### Multi-scale imaging of epileptiform activity

High-speed confocal microscopy was used to monitor ictal-like activity in agar-embedded larval zebrafish (Fig. 1*A*) with neuronal expression of GCaMP (*neurod1:GCaMP6f*; Fig. 1*B*). Larvae were immobilized in agar and imaged using a 5× (brain-wide) or 20× (single-cell) objective. For brain-wide studies, the confocal plane of view was adjusted to include forebrain (contains pallium and habenula), optic tectum (contains neuropil and stratum periventriculare; SPV), cerebellum and hindbrain (Fig. 1*D*,*E*). ROIs were drawn manually over anatomic areas corresponding to major brain regions, and fluorescence changes over time (ΔF/F) were extracted from time-lapse movies acquired at 20–30 Hz (Fig. 1*E*,*F*). In all PTZ-exposed larvae (*n* = 43), we observed large calcium transients at a frequency of one to two events per min, which occurred synchronously across all brain ROIs consistent with a generalized seizure classification (Fig. 1*E*; Movie 3). Large calcium transients were not seen in Control larvae bathed in embryo media (*n* = 9; Movie 1). As seizures are commonly associated with increased synchrony in neuronal populations (Penfield and Jasper, 1954; Jiruska et al., 2013), we also monitored calcium activity in SPV neurons within optic tectum microcircuits at single-cell resolution, which is facilitated by the mosaic *neurod1*-driven expression of GCaMP6f in this brain region (Fig. 1*F*). Exposure to PTZ elicited waves of calcium activity that showed a high degree of synchrony across all neurons (Movie 6).

Movie 1. Representative whole-brain imaging with 5× objective from control fish. Movie played at 5× speed. 10.1523/ENEURO.0041-19.2019.movie.1

**Figure 2.**
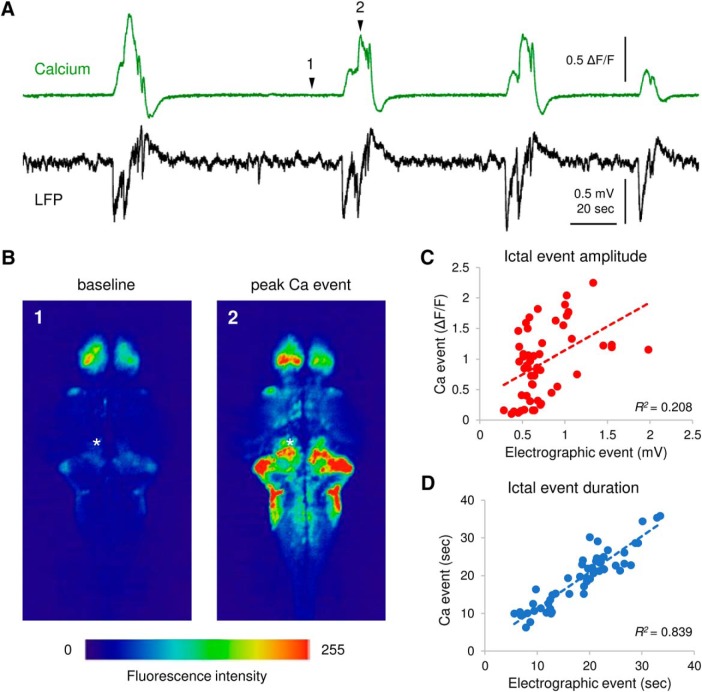
Correlation between LFP and calcium transients. ***A***, Representative simultaneous calcium traces (green) from the cerebellum and LFP (black) recorded from optic tectum/cerebellum with recurrent PTZ-induced ictal-like seizures. Scale bars as indicated in figure. ***B***, Fluorescence images of calcium activity during baseline and peak ictal-like event. The intensity of fluorescence is color coded as shown in the color bar. Events 1 and 2 as noted in ***A***. Asterisk indicates the LFP recording site. ***C***, ***D***, Correlation between LFP and calcium transients in ictal-like event amplitude (***C***) and duration (***D***). Corresponding correlation coefficient *R* ^2^ is indicated in figure; *n* = 54 events from 10 PTZ-treated larvae.

To determine whether synchronous calcium events correspond to electrographic discharges, we simultaneously monitored LFPs with an electrode placed in optic tectum SPV or cerebellum (as noted in Fig. 2*B*) in PTZ-treated larvae. Long-duration multi-spike ictal-like discharges coincided with large-amplitude calcium transients in every recording (Fig. 2*A*). The correlation between whole-brain calcium transient amplitude and LFP amplitude was low (*R* ^2^ = 0.208, *n* = 54 events from 10 PTZ-treated larvae; Fig. 2*C*). The relationship between whole-brain calcium transient duration and electrographic event duration was approximately linear (*R* ^2^ = 0.839, *n* = 54 events from 10 PTZ-treated larvae; Fig. 2*D*).

### Brain-wide network synchronization

**Figure 3.**
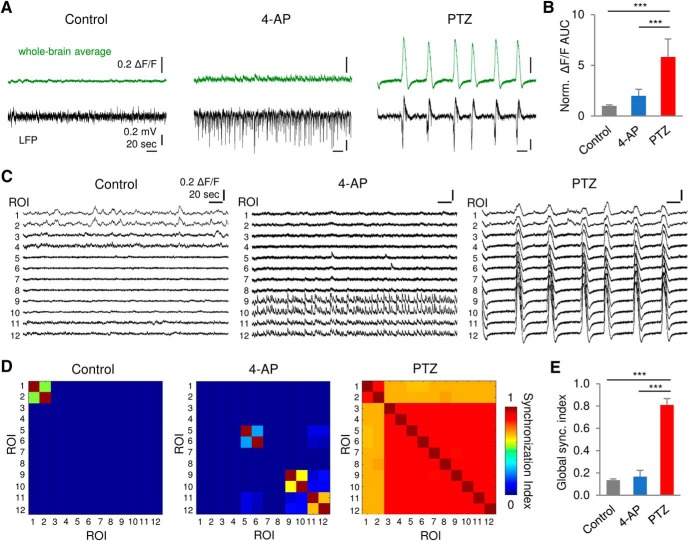
Brain-wide network synchronization. ***A***, Representative whole-brain average fluorescence changes (ΔF/F; green) and LFP (black) recorded from control, 4-AP, and PTZ-treated larvae. ***B***, Comparison of normalized AUC of fluorescence changes (norm. ΔF/F AUC). ***C***, Representative fluorescence changes of each brain region under different conditions. ROIs refer to Figure 1*E*. ***D***, Correlation matrices of whole-brain network activity for representative fish. ***E***, Comparison of global synchronization (sync.) index. Scale bars as indicated in figure; *n* = 4, 7, and 7 fish for control, 4-AP, and PTZ condition, respectively. Error bars indicate SD. Statistical significance is indicated as ***p < 0.001.

To investigate characteristics of synchronization, we analyzed brain-wide network dynamics in hyperexcitable 4-AP-treated and epileptic PTZ-treated zebrafish. Age-matched untreated larvae were studied as Controls. 4-AP and PTZ-treated larvae showed distinct activity patterns in both whole-brain average fluorescence changes and LFP (Fig. 3*A*). 4-AP-treated larvae were characterized by high-frequency short-duration spikes in LFP and frequent low-amplitude calcium transients. In contrast, PTZ-treated larvae consistently exhibited recurrent high-amplitude long-duration ictal-like events in both LFP and calcium traces, and significantly increased area under curve (AUC) in fluorescence changes compared with control and 4-AP-treated larvae (*p* < 0.001, one-way ANOVA; *n* = 4, 7, and 7 fish for control, 4-AP, and PTZ condition, respectively; Fig. 3*B*).

Next, we analyzed the activity of subregions within the larval whole-brain network. Representative fluorescence traces of each brain region (ROI numbering refers to Fig. 1*E*) and corresponding correlation matrices are shown in Figure 3*C*,*D*. Larvae exposed to 4-AP showed frequent short-duration and low-amplitude calcium transients in cerebellum and hindbrain regions only. In contrast, PTZ-treated larvae showed widespread high-amplitude and long-duration calcium transients across the entire nervous system, and significantly increased brain-wide synchronization compared with Control and 4-AP-treated larvae (*p* < 0.001, one-way ANOVA; *n* = 4, 7, and 7 fish for control, 4-AP, and PTZ condition, respectively; Fig. 3*E*). Whole-brain activity of control, 4-AP, and PTZ-treated fish are exemplified in Movies 1, 2, 3, respectively.

Movie 2. Representative whole-brain imaging with 5× objective from 4-AP fish. Movie played at 5× speed. 10.1523/ENEURO.0041-19.2019.movie.2

Movie 3. Representative whole-brain imaging with 5× objective from PTZ fish. Movie played at 5× speed. 10.1523/ENEURO.0041-19.2019.movie.3

### Seizure propagation

**Figure 4.**
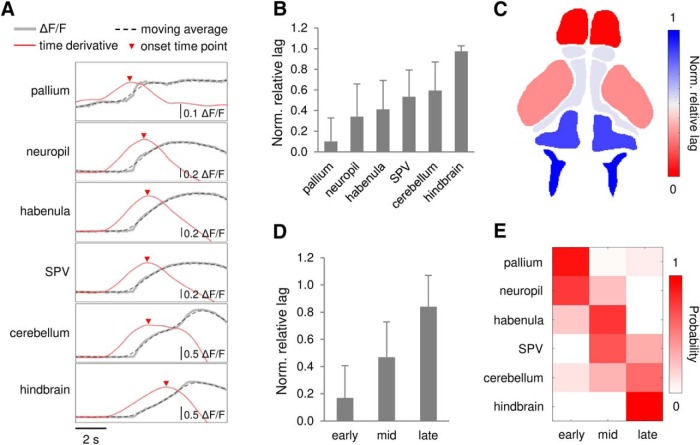
Seizure propagation across the whole-brain. ***A***, Representative calcium trace for each brain region within an PTZ-induced ictal-like event. Event onset time points were determined by the local maxima of the time derivative of moving average (of 50 data points) of ΔF/F. Scale bars as indicated in figure. ***B***, Plot of normalized (norm.) relative lag from the earliest onset. ***C***, Color-coded onset time mapping in a representative fish brain (average of four ictal events). ***D***, Normalized relative lag for three quantile groups (first third early onset, second third middle onset, and third third late onset). ***E***, Probability of brain regions falling into each quantile group. χ^2^ test X2(10) = 375.1, *p* < 0.001. Error bars indicate SD; *n* = 30 events from 7 PTZ-treated larvae.

To determine brain regions recruited during seizure initiation and propagation, we analyzed calcium changes in multiple brain regions (Fig. 1*E*) during PTZ exposure (*n* = 30 events from 7 PTZ-treated larvae). Figure 4*A* shows representative fluorescence traces of each brain region during an ictal-like event break-in. The red trace is the time derivative of ΔF/F moving average of 50 nearby data points, and the local maxima position corresponds to the onset time point of the brain region. We calculated the relative lag of each brain region and normalized it to the earliest-to-latest onsets interval. Arranging the normalized relative lag of brain regions revealed a coarsely preserved temporal ordering across ictal-like seizure events (Fig. 4*B*). Figure 4*C* shows a color-coded onset time mapping from a representative PTZ-induced ictal-like seizure events in one zebrafish (average of four ictal-like events). We then divided the normalized relative lags within one ictal-like event into three quantile groups: early, middle and late (Fig. 4*D*), and calculated the probability of each brain region falling into each temporal group. This analysis revealed a conserved propagation pattern from the anterior-to-posterior brain (χ^2^ test X2(10) = 375.1, *p* < 0.001; *n* = 30 events from seven PTZ-treated larvae; Fig. 4*E*).

### Neuronal ensembles in optic tectum microcircuits

**Figure 5.**
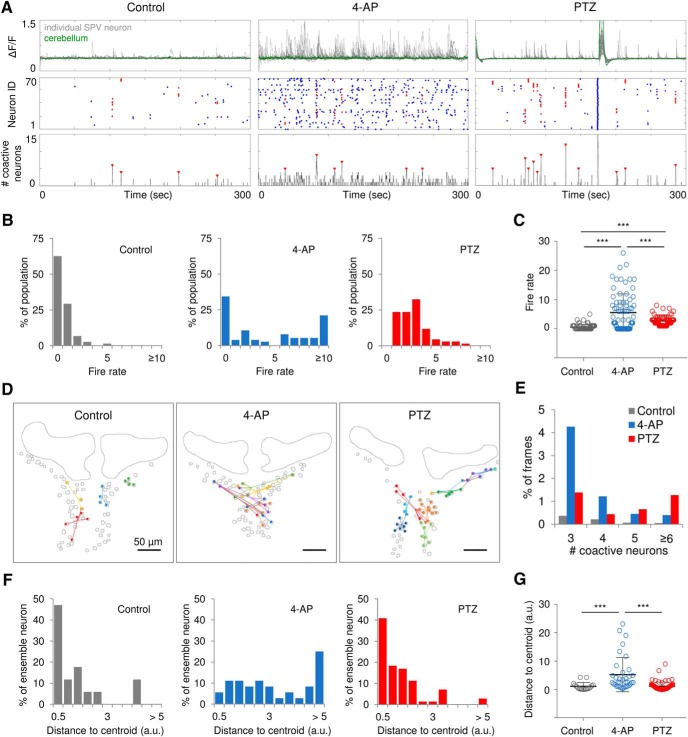
Neuronal ensembles in optic tectum microcircuits. ***A***, Representative calcium traces (gray, individual SPV neuron; green, cerebellum), neuron activation raster plot (red, ensemble neurons) and number of coactive neurons (ensembles were indicated by red arrow) of control, 4-AP, and PTZ-treated fish. Cerebellum activity was shown as an indicator of ictal-like events that involve all brain regions. ***B***, ***C***, Histogram (***B***) and distributions (***C***) of the neural firing rate (event count per 5 min); *n* = 75, 76, and 68 neurons for control, 4-AP, and PTZ-treated fish, respectively. ***D***, Spatial mapping of ensembles depicted in ***A***. Dots in the same color represent coactive neurons, and lines are distances from ensemble neurons to the corresponding ensemble centroid. Each ensemble is indicated by a color. Scale bars as indicated in figure. ***E***, Distribution of coactive neuron numbers as percentage of frames (6000 frames for each recording). ***F***, ***G***, Histogram (***F***) and distributions (***G***) of distances from ensemble neurons to the corresponding ensemble centroid; *n* = 17, 36, and 42 ensemble neurons for control, 4-AP, and PTZ-treated fish, respectively. Error bars indicate SD. Statistical significance is indicated as ***p < 0.001.

Next, we investigated network dynamics of the optic tectum microcircuit at single-neuron resolution. We analyzed individual SPV neuronal activity during interictal periods in representative control, 4-AP, and PTZ-treated fish, since ictal-like seizure events involve over 90% of the neuronal population in the field of view. Cerebellum activity, where baseline *neurod1:GCaMP6f* expression is prominent (Fig. 1*B*), was also extracted as a reference of ictal-like events that generalize to the entire nervous system. Based on binary event data generated from fluorescence changes with an automatic event detection algorithm (Patel et al., 2015), we constructed raster plots of neuronal activity, and then used a sliding window technique to generate a coactive neuron number time series (Fig. 5*A*). Ensemble events, namely a statistically significant number of coactive neurons compared with surrogate datasets, are marked by red arrows (Fig. 5*A*, bottom panel), and corresponding coactive neurons are colored red in the raster plot (Fig. 5*A*, middle panel). Chemoconvulsant (4-AP or PTZ) exposed larvae showed more frequent ensemble event occurrence compared with Controls. The neural firing rate (i.e., number of events in a 5-min recording epoch) distribution shows that both drug exposures increase excitability within the optic tectum network (Fig. 5*B*). For control fish, most neurons were inactive and firing at a low rate during the recording epoch. For 4-AP-treated fish, about half of the neurons exhibit firing rates between 1 and 10 events per 5 min, with ∼20% of neurons firing ≥10 events per 5 min. For PTZ-treated fish, no neurons were silent and firing rates were between 1 and 8 events per 5 min. Neurons in PTZ-treated fish showed significantly higher average firing rates than control fish, and neurons in 4-AP-treated fish had significantly higher average firing rate than both control and PTZ-treated fish (*p* < 0.001, one-way ANOVA; *n* = 75, 76, and 68 neurons for control, 4-AP, and PTZ-treated fish, respectively; Fig. 5*C*).

We then analyzed the spatial properties of neuronal ensembles. Representative spatial distribution plots of these ensembles (as noted in Fig. 5*A*) onto the optic tectum are shown in Figure 5*D*. Ensembles were detected in all control, 4-AP, and PTZ groups. Figure 5*E* shows the number of coactive neuron distributions within these ensembles. Control fish data showed few frames with coactive neurons (6000 frames for each recording). While 4-AP-treated fish showed more frames with small coactive neuron groups (number of coactive neurons <5), consistent with overall network hyperexcitability. PTZ-treated fish had more frames with larger coactive neuron groups (number of coactive neurons ≥5) than either Control or 4-AP-treated fish. In an epileptic network that is able to generate ictal-like events (i.e., PTZ-treated fish), spatially distributed ensembles are composed of larger groups of neurons.

The distance between ensemble centroid and each neuron within an ensemble also showed differences between groups (Fig. 5*F*,*G*). Ensembles observed in hyperexcitable 4-AP-treated fish network showed significantly larger spatial extent than in control or PTZ-treated fish (*p* < 0.001, one-way ANOVA; *n* = 17, 36, and 42 ensemble neurons for control, 4-AP, and PTZ-treated fish, respectively). Network dynamics of optic microcircuits are exemplified in Movie 4, 5, 6 for control, 4-AP, and PTZ-treated fish, respectively.

Movie 4. Representative imaging in optic tectum microcircuits with 20× objective from control fish. Movie played at 5× speed. 10.1523/ENEURO.0041-19.2019.movie.4

Movie 5. Representative imaging in optic tectum microcircuits with 20× objective from 4-AP fish. Movie played at 5× speed. 10.1523/ENEURO.0041-19.2019.movie.5

Movie 6. Representative imaging in optic tectum microcircuits with 20× objective from PTZ fish. Movie played at 5× speed. 10.1523/ENEURO.0041-19.2019.movie.6

## Discussion

Here, we combined sensitive GCaMPs and high-speed multi-scale monitoring of brain activity to characterize network dynamics in the CNS of larval zebrafish, following chemoconvulsant treatment. Our experiments revealed that ictal-like seizures are characterized by synchronous activation of the whole-brain network involving all major brain regions and propagate rapidly in a preserved temporal ordering: from anterior-to-posterior brain. PTZ-induced seizure and 4-AP-induced non-seizure hyperexcitability models showed distinct network dynamics in both whole-brain network and optic tectum microcircuits. Analysis of individual neuronal activities during interictal periods revealed neuronal ensembles in optic tectum, which demonstrated distinct properties in epileptic versus normal brain.

Optical monitoring in larval zebrafish provides a unique opportunity to map neuronal activity in a brain-wide manner. These measurements are not possible with electrophysiological approaches and led to spatiotemporal analysis of seizure initiation and propagation across the zebrafish CNS. Previous imaging studies using an acute PTZ seizure model in zebrafish focused on optical mapping of seizure propagation (Tao et al., 2011) or brain activities coincident with tail movements (Turrini et al., 2017), using mathematical modeling to reveal network connectivity changes during seizures (Rosch et al., 2018), and functional profiling of drug action in multiple brain regions (Winter et al., 2017). Generalized brain-wide activation during ictal-like seizure events was reported in these studies using acquisition speed ranges from 1 to 20 Hz. Here, using a fast spinning-disk confocal we observed similar patterns of brain activity with acquisition speeds up to 30 Hz and discovered a reproducible anterior-to-posterior propagation pattern of rapidly generalized optical seizures (Fig. 4).

To confirm the correlation between electrographic and optical activity, we acquired LFP and calcium imaging data simultaneously (Fig. 2). A high degree of correlation was observed in event duration but not in event amplitude (Fig. 2*C*,*D*). This is not surprising, as LFP event amplitude is dependent on electrode tip position in the brain relative to sources and sinks of current (Buzsáki et al., 2012), and calcium signal amplitude is linked to focal plane and GCaMP expression intensity variabilities between fish. Although both methods report generalized ictal-like seizure events consistently, LFP and calcium imaging do not measure identical activity. For example, single and multi-unit background activity shown in LFP recording in Control fish was not seen in corresponding calcium trace (Fig. 3*A*, left); 4-AP fish showed frequent high-amplitude spikes in LFP (Fig. 3*A*, middle), but little to no fluorescence change was observed in the recording region (optic tectum/cerebellum; ROIs 7–10 in Fig. 3*C*, middle). These observations reveal the relative advantages and disadvantages of each approach. Compared with LFP recording, GCaMP-based imaging provides superior spatial resolution but inferior detection sensitivity and speed (Chen et al., 2013), which may not effectively detect sub-threshold synaptic transmission or capture fast spikes seen in LFP recordings. Conversely, LFP recordings are limited to a single recording site in the brain.

4-AP has been reported to induce ictal-like discharges *in vitro* (Galvan et al., 1982; Avoli et al., 2002) and *in vivo* (Szente and Pongrácz, 1979; Peña and Tapia, 2000), but it is not a robust state in all conditions (Perreault and Avoli, 1991, 1992; Mattia et al., 1993; Zhu et al., 2008; Avoli and Jefferys, 2016). In this work, ictal-like seizure events were not consistently observed in 4-AP-exposed zebrafish larvae at a concentration of 4 mM, except a fatal long-lasting burst after prolonged exposures (data not shown), which is consistent with other zebrafish-based imaging studies (Winter et al., 2017). 4-AP-induced neuronal hyperexcitability revealed by LFP recordings (Fig. 3) and imaging of microcircuit dynamics (Fig. 5) was also in line with previous reported behavioral manifestations in 4-AP-treated zebrafish (Kumar et al., 2016) and provided us with an hyperexcitable state that was not complicated by the presence of ictal-like activity.

When examining neuronal dynamics in optic tectum microcircuits, we detected ensembles of coactive neurons during interictal periods and showed that, in PTZ-treated fish, ensembles were spatially localized, while in 4-AP-treated fish, ensembles were smaller (contained less neurons) and dispersed (Fig. 5). Neuronal ensembles could also be detected in Control fish, but the ensemble event occurrence was rare. Similar observations of functional clusters were reported in calcium imaging studies from hippocampal slices prepared from chronically epileptic mice (Muldoon et al., 2013). Interestingly, “microseizures,” which are discharges from highly confined neuron groups within ictal onset zones, have been observed in intracranial recordings from microelectrode arrays in human patients with partial epilepsy (Schevon et al., 2008, 2010; Stead et al., 2010). Although precise spatiotemporal information of micro-discharge clusters is absent in these studies, it raises an intriguing hypothesis that ictal networks are composed of sparse pathologic microdomains that can generate microseizures, and the spreading of microseizures results in ictal seizures (Stead et al., 2010). Here we employed a well-established acute zebrafish seizure model and high-speed calcium imaging with single-cell resolution to map neural activations. This strategy revealed neuronal ensembles in an ictal network (the optic tectum microcircuit), which possibly correspond to human microseizure domains. Future studies using three-dimensional light sheet imaging (Ahrens et al., 2013b; Portugues et al., 2014; Dunn et al., 2016) with enhanced scanning speed could improve the neuron sampling and provide an even better understanding of the role of neuronal ensembles in seizure generation and propagation.

In summary, we established a zebrafish-based calcium imaging platform to evaluate network dynamics underlying recurrent seizures, including network synchronization, seizure propagation and neuronal ensembles within local microcircuits. Future work with improved imaging techniques to explore the interplay between micro-ensembles and macro-seizures, combined with zebrafish models mimicking genetic forms of epilepsy, will provide insights into seizure prediction and suppression.

## Synthesis

Reviewing Editor: Liset Menendez de la Prida, Instituto Cajal CSIC Decisions are customarily a result of the Reviewing Editor and the peer reviewers coming together and discussing their recommendations until a consensus is reached. When revisions are invited, a fact-based synthesis statement explaining their decision and outlining what is needed to prepare a revision will be listed below. Note: If this manuscript was transferred from JNeurosci and a decision was made to accept the manuscript without peer review, a brief statement to this effect will instead be what is listed below.

Manuscript transfered from JN after reviewing. Authors have made a good job in addressing issues raised by the previous reviewers at JN. I have personally checked their answers to their points and found the current version suitable for eNeuron. This is an interesting paper that combines genetically-encoded calcium indicators with whole brain imaging in zebrafish larvae to monitor pharmacologically induced activity patterns. The paper is relevant with potential application in anti-seizure drug screening. I therefore accept this paper for publication.

For the records, here is a synthesis of the issues raised in the reviewing version at JN.

Major comments:

From looking at the data, I cannot agree that the current method for detecting the regional onset of an ictal-like event relates to the actual regional onset of the event. Looking at Fig 4, there is a clear upward “notch” in the df/f signal at the onset of the event, and the since the 50 point averaging smooths the signal, the point of steepest ascent does not depict the “onset” that one would naturally ascribe. It seems that the region is slow to fully participate in the event however, given the soft shape of the df/f rise, which I assume is due to the large spatial coverage of the region and a non uniform start of the event within the region? Perhaps it would be more accurate to look at the peak point of participation (max amp of df/f within the event) and assess seizure spreading based on this? Given the shape of the signals, I cannot imagine a biological interpretation of the maximum of the time derivative as is currently calculated.

I find the use of the phrase “neuronal ensemble” to refer both to the time point, and neurons involved in time points containing a statistically significant increase in neuronal firing to be rather confusing. It would be more clear to define “events” in time and then discuss the neuronal population of said events.

Was any sort of cluster analysis applied to determine if “neuronal ensembles” reflected the co-activation of smaller ensembles as in Muldoon et al, 2013? At least in the PTZ data, it looks like certain “neuronal ensembles” contain the same neurons in different events (small ensembles in Fig 5A); do multiple small ensembles combine in other larger events?

Is there a relationship between the neuronal populations involved in events in 4-AP data and PTZ data? For example, if neurons 3,4,5 fired together in a neuronal ensemble in 4-AP events, did they also fire together in PTZ events? This is somewhat related to my comment above about the larger PTZ events potentially being comprised of multiple smaller ensembles. Given the relationship between spatial properties of the ensembles under the two conditions this seems not likely, but it would be worth investigating in more detail.

I noted with interest that the large electrographic discharges in 4-AP were generally not associated with very large Ca signal, consistent with others' observations in rodent tissue that this, at least initially, triggers primarily interneuronal activation (eg various works by Avoli & de Curtis, mainly of electrophysiological recordings, but relevant is Parrish et al 2018, J.Neurophys, which also examined 4AP activity using GCaMP expressing mice) - this relationship to the rodent work should probably be discussed. In fact, 4-AP did not appear to trigger full seizures, which was perhaps counter to expectation - full seizures were only really seen with the PTZ. They described a consistent anterior to posterior propagation pattern in these PTZ induced seizures. The 4-AP only showed “ensemble” activity, albeit at a higher rate than in untreated control preparations, and no description of any propagation was provided of these - I'm guessing that they did not propagate, but were “standing waves”, but could this be clarified, please?

The lack of seizures in the 4-AP is a shame - I would have expected these. It means that the only seizures are essentially from a disinhibition model, which is likely to show the least interesting neuronal dynamics. Disinhibition models in rodent tissue also propagate widely and with great speed, whereas far more interesting dynamics generally arise from the interplay between inhibitory elements in the network. Having a model that showed both the interesting ensembles and also transitions into seizures may have had a lot more substance, since it would allow one to examine pathways into the seizures. One benefit of being able to image the entire brain is that it does allow one to hone in on where the seizures start. This appears to be the pallium, yet the analysis of ensembles focussed largely on a different brain area, namely the tectum.

A final major concern is that this felt like it was primarily a presentation of new technique, and there was not a whole lot of new neurobiology. I also thought that the background pitch was a little disingenuous in its claims that previous imaging / recordings failed to capture widespread network dynamics. It should be noted that the MEA studies use arrays that are larger than the entire larval zebrafish brain. Furthermore, the Carandini lab (Rossi et al, 2017, Nat Comm) has used a similar approach to visualise epileptic propagation across wide fields of view in mice. There were no references generally about GECIs or GCaMP.

Minor comments:

How are the calcium event and electrographic event amplitude and duration defined?

The reference for calculating the global synchronization index in the “Network Synchronization” section appears to be incorrect. Please better define this measure and include the proper reference.

It seems that the “correlation matrices” of Figure 3E actually pairwise synchronization matrices computed using the phase synchronization metric.

In terms of presentation, the manuscript was generally well written, and I did not find many mistakes. A handful of points might be clarified. In figure 2C, were these mixed data from the two models, or all PTZ (I'm guessing, since 4-AP only produced low level activation). Likewise, in figure 4, is all the data from PTZ treated animals too (again, I'm guessing because these are ictal)?

Figure 5 - the legend indicates that these are analyses of optic tectum activity, yet panel A is labelled as “cerebellum”.

I was slightly surprised that the 4-AP appeared to show larger ensembles than the PTZ, but was this because larger PTZ events invariably escalated to be full seizures?

The terminology did not seem quite right to me, with the 4-AP data being described as a model of hyperexcitability. I'd have said that the PTZ also fell under that umbrella term. This is stated clearly early in the results section, but elsewhere, when the authors refer to hyperexcitable states, it seems odd not to include the PTZ data in these, and I know that is not what is intended here. I just felt that this introduced potential for confusion, and the authors might care to rethink their terminology.
